# Evaluation of the European Committee on Antimicrobial Susceptibility Testing Guidelines for Rapid Antimicrobial Susceptibility Testing of *Bacillus anthracis*-, *Yersinia pestis*- and *Francisella tularensis*-Positive Blood Cultures

**DOI:** 10.3390/microorganisms9051055

**Published:** 2021-05-13

**Authors:** Ohad Shifman, Tamar Aminov, Moshe Aftalion, David Gur, Hila Cohen, Elad Bar-David, Ofer Cohen, Emanuelle Mamroud, Haim Levy, Ronit Aloni-Grinstein, Ida Steinberger-Levy, Shahar Rotem

**Affiliations:** 1 The Department of Biochemistry and Molecular Genetics, Israel Institute for Biological Research, Ness Ziona 7410001, Israel; tamara@iibr.gov.il (T.A.); moshea@iibr.gov.il (M.A.); gurd@iibr.gov.il (D.G.); hilac@iibr.gov.il (H.C.); oferc@iibr.gov.il (O.C.); emmym@iibr.gov.il (E.M.); ronitag@iibr.gov.il (R.A.-G.); idasl@iibr.gov.il (I.S.-L.); 2 The Department of Infectious Diseases, Israel Institute for Biological Research, Ness Ziona 7410001, Israel; eladb@iibr.gov.il (E.B.-D.); haiml@iibr.gov.il (H.L.)

**Keywords:** blood culture, *Bacillus anthracis*, *Yersinia pestis*, *Francisella tularensis*, rapid antimicrobial susceptibility testing, RAST, EUCAST, Etest, disc diffusion, AST

## Abstract

Rapid determination of bacterial antibiotic susceptibility is important for proper treatment of infections. The European Committee on Antimicrobial Susceptibility Testing (EUCAST) has recently published guidelines for rapid antimicrobial susceptibility testing (RAST) performed directly from positive blood culture vials. These guidelines, however, were only published for a limited number of common pathogenic bacteria. In this study, we evaluated the applicability of these guidelines to three Tier 1 bioterror agents (*Bacillus anthracis*, *Yersinia pestis* and *Francisella tularensis*) that require prompt antibiotic treatment to mitigate morbidity and mortality. We used spiked-in human blood incubated in a BACTEC™ FX40 system to determine the proper conditions for RAST using disc-diffusion and Etest assays. We found that reliable disc-diffusion inhibition diameters and Etest MIC values could be obtained in remarkably short times. Compared to the EUCAST-recommended disc-diffusion assays that will require adjusted clinical breakpoint tables, Etest-based RAST was advantageous, as the obtained MIC values were similar to the standard MIC values, enabling the use of established category breakpoint tables. Our results demonstrate the promising applicability of the EUCAST RAST for *B. anthracis*-, *Y. pestis*- or *F. tularensis*-positive blood cultures, which can lead to shorter diagnostics and prompt antibiotic treatment of these dangerous pathogens.

## 1. Introduction

*B. anthracis*, *Y. pestis* and *F. tularensis* are three pathogens that are categorized by the U.S. Centers for Disease Control and Prevention (CDC) as Tier 1 biological select agents [1]. Without proper treatment, high rates of mortality are observed within a short period following infection [2,3,4,5]. These characteristics led to the recognition of these pathogens as potential biological threat agents. Anthrax, the disease caused by *B. anthracis*, can appear in three major forms: cutaneous, gastrointestinal and inhalational. Although a vaccine was developed against *B. anthracis*, vaccination is not routinely given, and antibiotic therapy is the treatment of choice following exposure. Without treatment, anthrax progresses rapidly and can be fatal in most cases [4,5,6]. *Y. pestis* is the etiological agent of plague, a severe and fatal disease. This disease has several major forms, bubonic, pneumonic and septicemic, depending on the route of exposure [2,5]. In the absence of proper antibiotic treatment administered within 18 to 24 h of symptom onset, high rates of mortality are observed. *F. tularensis* is the etiological cause of tularemia. Tularemia can also appear in several major forms of illness: ulceroglandular, glandular, oculoglandular, oropharyngeal, typhoid and pneumonic. Without proper antibiotic treatment, tularemia can result in mortality rates of up to 60%, and antibiotic therapy can reduce these rates to 1% [3,5]. As antibiotic treatment is the major therapeutic tool for these acute and rapidly progressing diseases, there is a concern that antibiotic resistance may result either from natural emergence or from deliberate engineering for a bioterror purpose [7,8,9,10,11,12,13,14,15]. Thus, there is a necessity for rapid antibiotic susceptibility testing that will offer the correct treatment for these diseases to reduce mortality rates, length of hospital stay and hospitalization costs [16,17].

Blood cultures (BCs) are the main source for bacterial isolation for antimicrobial susceptibility testing (AST) purposes. Conventional AST from positive BCs requires a concentration-defined suspension of bacteria, which is achieved by plating and incubating a sample of the blood culture on agar plates [18]. Incubation on agar plates also reduces possible growth inhibition that may be caused by blood components, antibiotics administered to the patient or BC media supplementations, which may affect the AST results. However, the isolation step is time consuming, especially in bacteria that grow slowly in vitro, such as *Y. pestis* and *F. tularensis*. Over the years, attempts have been made to develop RAST assays [19,20,21,22]. However, most of these methods are not standardized, hampering their acceptance in clinical laboratories. Recently, the EUCAST and the Clinical and Laboratory Standards Institute (CLSI) have proposed standard RAST assays based on shortened disc-diffusion assays, which are conducted after plating samples obtained directly from BC vials and interpreted by using dedicated susceptibility breakpoint interpretation tables [23,24,25,26]. However, these RAST guidelines were validated and published for only a limited number of common life-threatening bacteria that can easily acquire antibiotic resistance (*Escherichia coli*, *Klebsiella pneumoniae*, *Pseudomonas aeruginosa*, *Acinetobacter baumannii*, *Staphylococcus aureus*, *Enterococcus faecalis*, *Enterococcus faecium* and *Streptococcus pneumoniae*) [26]. Other studies have shown the feasibility of these approaches for additional bacteria, but also highlighted the importance of determining whether this direct method is implementable for each bacterial–antibiotic combination [23,25,27,28,29,30,31,32,33,34].

In this study, we examined the applicability of the RAST approach to *B. anthracis*, *Y. pestis* and *F. tularensis*, three Tier 1 select biological agents, which have not been evaluated before. As bacteremia is evident for all three diseases caused by these bacteria, establishing a standard RAST directly from BCs can accelerate proper treatment and recovery. We used human blood cultures spiked with either *B. anthracis*, *Y. pestis* or *F. tularensis*. We then plated samples taken directly from the BCs for determination of the optimal conditions for proper RAST using disc-diffusion and Etest assays. We found that by using Etest-based RAST, reliable MIC results can be obtained for the recommended therapeutic antibiotics, following 6 to 8 h, 18 to 24 h and 24 to 30 h of incubation for *B. anthracis*, *Y. pestis* and *F. tularensis*, respectively. These results suggest that RAST can be successfully applied for these Tier 1 select agents.

## 2. Materials and Methods

### 2.1. Bacterial Strains, Growth Conditions and Colony Forming Unit Determination

*B. anthracis* Vollum pXO1^-^ pXO2^-^ spores [35] were germinated to vegetative form in terrific broth [36] at 37 °C for 30 min before use. The *Y. pestis* EV76 vaccine strain [37] was routinely grown on brain heart infusion agar (BHIA, BD Cat #241830) plates at 28 °C. The *F. tularensis* live vaccine strain (LVS, ATCC 29684) was grown on Cystine Heart Agar (CHA) plates (BD Cat #247100) enriched with 1% (*w*/*v*) hemoglobin (BD, Cat #212392) at 37 °C. Colony forming units (CFUs) were counted by drop plating, in triplicate, 10 µL from serial tenfold culture dilutions in sterile Dulbecco’s phosphate-buffered saline (PBS, Biological Industries Cat #02-023-1A) on Muller Hinton agar (MHA, BD Cat #225250), BHIA and CHA plates for *B. anthracis*, *Y. pestis* and *F. tularensis*, respectively.

### 2.2. Antibiotics

Antibiotics for disc-diffusion AST assays were purchased from BD and Oxoid; Etest strips were purchased from bioMerieux, Marcy l’Etoile, France. The antibiotic concentrations, catalog numbers and abbreviations used in this study are given in Table 1. The antibiotics were stored according to the manufacturers’ instructions until use.

### 2.3. Antimicrobial Susceptibility Testing

Standard AST was performed by spreading 125 µL of bacterial suspension (~10^8^ CFU/mL) on 90 mm agar plates (MHA for *B. anthracis* and *Y. pestis*, CHA for *F. tularensis*) using a Drigalski spatula and application of 2–3 discs or 1–2 Etest strips on each of the swabbed plates. The plates were incubated at 35 °C, and standard values were determined at 18 and 20 h (*B. anthracis*) or 24 h (*Y. pestis*). *F. tularensis*-seeded plates were incubated at 35 °C with 5% CO_2_ for 48 h. RAST was performed by spreading 125 µL of culture sampled from a positive BC vial on 90 mm agar plates using a Drigalski spatula and applying antibiotic discs or Etest strips on the swabbed plates. The plates were incubated under the same conditions as used for the standard AST.

### 2.4. MIC and Inhibition Zone Diameter Determination

For the Etest assays, the MIC values were determined by reading the scale values on the strips at the intersection of the growth inhibition zone (from the front of the plate with the lid removed) according to the manufacturer’s guidelines. MIC values were rounded up to the nearest twofold antibiotic concentration. For the disc-diffusion tests, the inhibition zone diameters were measured from the front of the plate with the lid removed according to EUCAST guidelines.

## 3. Results

Recently, EUCAST published guidelines for the rapid determination of antibiotic susceptibility directly from BCs. The improvement in the assay duration was achieved by addressing two aspects. First, bacteria were plated on agar plates directly from the BC vials without prior isolation, enrichment and quantification of the bacterial inoculum, steps that are time consuming and labor intensive. Second, the antibiotic inhibition zones were read at earlier times (4 to 8 h) compared to the standard reading times (18 to 20 h). We sought to evaluate whether these shortcuts could also be applied to *B. anthracis*, *Y. pestis* and *F. tularensis*, three Tier 1 select agents on the CDC list for which RAST can be beneficial. We first analyzed the applicability for *B. anthracis*. A similar analysis for *Y. pestis* and *F. tularensis* will be presented thereafter.

### 3.1. Bacillus anthracis

#### 3.1.1. Early Reading of AST Results

First, we wanted to determine the earliest time points for which reliable AST results can be obtained for *B. anthracis*. The reading times are affected by the ability to visually examine, by an unaided eye, the growth of the bacteria and, hence, the determination of inhibition zones and the change in the inhibition zone over time. To this end, we conducted disc-diffusion assays with a panel of antibiotics, including the CLSI- and EUCAST-recommended antibiotics (CIP, TET and BEN) and five additional potential therapeutic antibiotics (Table 2). Using a suspension of germinated *B. anthracis* spores at the standard inoculum concentration of 1 × 10^8^ CFU/mL, we determined the inhibition zone diameters at various time points, including early reading time points, as suggested by the EUCAST RAST guidelines (6 to 8 h) and the standard reading time points (18 to 20 h). Distinct inhibition edges were evident as early as 6 h of incubation for all the tested antibiotics. However, for most of the tested antibiotics, the inhibition zone diameters differed between the shortened reading times (6 to 8 h) and the recommended reading times for standard tests (18 to 20 h). These results suggest that reading results at shortened time points is feasible, but specific susceptibility breakpoint criteria should be determined for the shortened incubation for RAST implementation. We also tested the possibility of determining MIC values using Etest strips for additional antibiotic substances (Table 3). For the disc-diffusion-based assay, the edges of the inhibition zone could be visible after 6 h of incubation. In these assays, however, the MIC values for most antibiotics (CIP, AMP, CHL, CLI and CLA) at early reading times (6 to 8 h) and standard reading times (18 to 20 h) were similar; thus, susceptibility category interpretation for RAST can be based on the standard breakpoint interpretation tables [38] or standard AST results. DOX, VAN and RIF were the only antibiotics in which their MIC values at shortened reading times were different from those obtained at the standard times; thus, even Etest-based RAST implementation will require dedicated breakpoint interpretation tables.

#### 3.1.2. Characterization of *B. anthracis* Growth in Human Blood Culture Vials

Before applying RAST to *B. anthracis*-grown BCs, we characterized the correlation between the inoculum concentration and the incubation time needed for the BCs to be flagged as positive. In addition, we determined the bacterial concentrations when BCs flagged positive. We spiked human blood with *B. anthracis* at a wide range of concentrations (1 × 10^1^ to 1 × 10^5^ CFU/mL) and transferred the spiked blood into BACTEC™ Plus Aerobic/F culture vials. The samples were then incubated in a BACTEC™ FX40 device until they were flagged as positive by the device. At this point, the bacterial concentrations were determined by CFU counting (Table 4).

Remarkably, despite the large difference in the initial *B. anthracis* concentrations, the bacterial concentrations at the alert were relatively similar and in a narrow range of 6.0 × 10^6^ to 2.9 × 10^7^ CFU/mL. This was a consequence of prolongation in the incubation time needed for the culture to turn positive at lower inoculum concentrations, with a range of 4.5 to 6.5 h needed for the highest-spiked inoculum of 1.0 × 10^5^ CFU/mL to 11 to 12 h for the lowest spiked inoculum of 1.0 × 10^1^ CFU/mL. We also examined the bacterial concentrations at the maximal postponement after the alert allowed by the EUCAST regulations before applying RAST (18 h of incubation in the device or 3 h at room temperature). In both cases, bacterial viability was maintained, as bacterial CFUs were at least as high as those at the alert, suggesting that RAST can be evaluated for these time frames.

#### 3.1.3. RAST Directly from Positive Blood Culture Vials

Finally, we wanted to evaluate the EUCAST method for RAST directly from *B. anthracis*-positive BC vials. According to the EUCAST guidelines, RAST can be performed 0 to 18 h after BCs have signaled positive, provided that during this period, the BC vials were not removed from the device. In cases where the vials are removed from the device and kept at room temperature, RAST can be performed up to 3 h thereafter. Accordingly, we examined the RAST performance at three time points: when the vials were flagged as positive, 18 h after they were positively flagged and were left in the device, and 3 h after they were positively flagged and were kept at room temperature. The antibiotics used were those analyzed before using the standard inoculum culture. For all the antibiotics tested, including ciprofloxacin (Table 5), tetracycline, penicillin G, amoxicillin–clavulanic acid, linezolid, moxifloxacin, levofloxacin and imipenem (Supplementary Tables S1–S7), the inhibition zone diameters obtained by RAST directly from positive BCs (“at the alert” columns) were similar to the diameters obtained for the standard culture at the corresponding reading times (“standard culture” columns).

As was observed for the standard culture, for most antibiotics, the diameters at the shortened reading times were different from those of the longer reading times. These results suggest that although RAST performed directly from BCs is feasible for *B. anthracis*, specific interpretation breakpoint tables should be established for shorter reading times. Postponement of the culture, either 18 h in the device or 3 h at room temperature, did not affect the inhibition zone diameters (Table 5, “18 h after the alert” and “3 h after the alert” columns). Thus, RAST can be performed according to the EUCAST time windows.

We also performed Etest assays directly from these BCs and compared the MIC values to those that we acquired previously using the standard inoculum (Section 3.1.1). For all antibiotics tested—ciprofloxacin (Table 6), doxycycline, ampicillin, clarithromycin, chloramphenicol, clindamycin, vancomycin and rifampicin (Supplementary Tables S8–S14)—there was good agreement between the MIC values obtained from BCs and the MIC values that were obtained by the standard AST. These results also hold true for vials kept 18 h in the device or 3 h at room temperature, the maximal postponement length windows allowed by EUCAST regulations. Therefore, our results suggest that Etest-based RAST can be applied directly from *B. anthracis*-positive BCs, starting from 6 h following plating.

### 3.2. Yersinia pestis

#### 3.2.1. Early Reading of AST Results

We first aimed to determine the shortest incubation time for which reliable AST results can be obtained for *Y. pestis*. Therefore, we performed disc-diffusion-based AST using a standard *Y. pestis* inoculum (~1 × 10^8^ CFU/mL originating from isolated colonies) and determined the inhibition zone diameters at different incubation times for a panel of potential therapeutic antibiotics (ciprofloxacin, tetracycline, gentamicin, chloramphenicol and trimethoprim–sulfamethoxazole). Distinct inhibition zone edges were evident only after 18 h of incubation for all tested antibiotics (Table 7).

For CHL and TRS, the inhibition zone diameters were similar between the recommended reading time (24 h) and the earlier time points, and therefore, the AST can be shortened for these antibiotics. For the other antibiotics (CIP, TET and GEN), the inhibition zone diameters varied slightly between the shortened 18-hour reading time and the standard reading time (24 h); thus, to shorten the AST, explicit breakpoint tables will be required for the earlier time points. We also checked the possibility of determining MIC values using Etest strips. The Etest MIC values were read at the same time points as for the disc-diffusion assay. Also for the Etest assays, growth inhibition zones were visible only after 18 h of incubation. However, in contrast to the disc-diffusion test, where only CHL and TRS had similar inhibition zone diameters at the 18 h and 24 h readings, Etest assays resulted in similar MIC values for all six tested antibiotics (Table 8). Thus, susceptibility breakpoints could be interpreted following a shortened 18-hour incubation using *Y. pestis* standard breakpoint tables, such as those published by the CLSI [38].

#### 3.2.2. Characterization of *Y. pestis* Growth in Human Blood Cultures

Next, we characterized the incubation time needed for *Y. pestis*-spiked BCs to be flagged as positive by the BACTEC™ FX40 system and determined the bacterial concentrations at that time point. For this purpose, we spiked human blood with *Y. pestis* bacteria at various concentrations (1.4 × 10^1^ to 2.7 × 10^3^ CFU/mL; Table 9) and transferred the spiked blood into BACTEC™ Lytic Anaerobic/F culture vials, which were then incubated in a BACTEC™ FX40 device until they were flagged as positive. BACTEC™ Lytic Anaerobic/F culture vials were used, as we found that *Y. pestis* grew faster in those vials than in aerobic vials.

As observed for *B. anthracis*, *Y. pestis* concentrations at the alert were similar (1 × 10^7^ to 5.6 × 10^7^ CFU/mL, “concentration at the alert” column), despite ~2 orders of magnitude difference in the concentrations of bacteria initially spiked in the vials. This was compensated by a longer incubation duration (from 20–22 h to 25–29 h) for BCs spiked with low bacterial concentrations. We also examined the bacterial concentrations following further incubation after the alert (18 h in the device or 3 h at room temperature). CFU counting showed that *Y. pestis*, similar to *B. anthracis*, maintained viability under those conditions, indicating that RAST can be performed in these time frames.

#### 3.2.3. RAST Directly from Positive Blood Culture Vials

Finally, we aimed to evaluate the applicability of EUCAST RAST directly from positive *Y. pestis* BC vials. We followed EUCAST guidelines and examined the possibility of performing RAST at three time points; when the BC bottles were flagged as positive, 18 h after they were flagged as positive and continued to incubate in the BACTEC™ FX40 device, and after they were flagged as positive and were kept at room temperature for an additional 3 h. The positive BCs were tested using the same panel of potential therapeutic antibiotics as previously tested using standard colony-suspended bacterial cultures. For all antibiotics tested, inhibition zone diameters could be observed by RAST performed on the positive BCs as early as 18 h (Table 10 for ciprofloxacin and Supplementary Tables S15–S18 for tetracycline, gentamicin, chloramphenicol and trimethoprim–sulfamethoxazole).

However, as there were differences in the inhibition zone diameters resulting directly from BCs compared to the diameters of standard conditions for some of the antibiotics, explicit breakpoint tables will be required for the implementation of RAST for *Y. pestis*-positive BCs. In contrast to the disc-diffusion-based AST, using Etest strips for RAST (Table 11 for ciprofloxacin and Supplementary Tables S19–S23 for doxycycline, chloramphenicol, trimethoprim–sulfamethoxazole, gentamicin and streptomycin) resulted in MIC values that were similar to those obtained under standard conditions, starting from 18 h of incubation. These results support the usage of breakpoint interpretation tables that were determined for *Y. pestis* under standard conditions for Etest-based RAST at all time points, starting from an 18-hour incubation.

### 3.3. Francisella tularensis

#### 3.3.1. Early Determination of AST Results

For *F. tularensis*, we first determined, for a panel of potential therapeutic antibiotics, the earliest time points for which reliable AST results can be obtained. For this purpose, we performed a disc-diffusion-based test using a standard *F. tularensis* suspension obtained from isolated colonies and determined the inhibition zone diameter at different time points. As *F. tularensis* does not grow on MHA, we used Muller Hinton Fastidious (MH-F) agar (purchased from two vendors), which is EUCAST’s recommended growth medium for fastidious bacteria. Unfortunately, this growth medium also did not properly support the growth of *F. tularensis*, impeding its use for AST assays. We, therefore, performed assays using CHA, a solid growth medium commonly used for *F. tularensis* growth and antimicrobial susceptibility tests [39]. Distinct inhibition zone edges were evident as early as 24 h following incubation for all the antibiotics tested—CIP, TET, CHL and GEN (Table 12).

However, for CIP, TET and GEN, the inhibition zone diameters differed between 24 h and the standard reading times (48 h). Hence, to shorten the reading times to 24 h, distinct breakpoint tables should be created. Reading the inhibition zone following a 30-hour and up incubation period, the inhibition zone diameters were similar to those observed at 48 h for all four tested antibiotics. When we used Etest strips to determine MIC values (Table 13), the MIC values at 24 h and at the standard reading time (48 h) were similar; thus, using Etest-based RAST, proper MIC values could be read as early as 24 h of incubation, and breakpoint determination could be obtained from standard breakpoint tables, such as those published by CLSI [38].

#### 3.3.2. Characterization of *F. tularensis* Growth in Human Blood Culture

Next, we analyzed the growth rates of *F. tularensis* in BC vials and the bacterial concentrations when the vials were flagged as positive. We spiked *F. tularensis* into human blood with a wide range of bacterial concentrations (Table 14, 2 × 10^1^ to 2.9 × 10^6^ CFU/mL) and transferred the spiked blood into BACTEC™ Plus Aerobic/F culture vials, which were then incubated in the BACTEC™ FX40 device until the vials were marked as positive. The bacterial concentrations were counted at the alert and, following EUCAST guidelines, after an additional 18 h of incubation in the device or after an additional 3 h of incubation at room temperature. As observed for *B. anthracis* and *Y. pestis*, the *F. tularensis* concentrations at the device alert were at a narrow range of ~1 order of magnitude (1.4 × 10^7^ to 3.6 × 10^8^ CFU/mL; “concentration at the alert” column) compared to the broad 5-orders-of-magnitude range in the inoculum concentrations.

Moreover, the incubation duration until the vials were marked as positive was negatively correlated with inoculum concentrations, and while ~1 day was needed for ~10^6^ CFU/mL spiked BC to become positive, 6 to 12 days were required for cultures spiked with 20 to 40 CFU/mL (“incubation duration until the alert” column). Further incubation of the BCs in the device or at room temperature (18 or 3 h, respectively) showed that bacteria were still growing under those conditions. Hence, it is applicable to initiate RAST with postponed BCs. Notably, for BCs containing low concentrations of *F. tularensis*, the growth rates were very slow, exceeding the 5-day default incubation protocol used by the device to flag BCs as negative; thus, false-negative results could be obtained when low levels of *F. tularensis* were present in the blood. Furthermore, when we assessed *F. tularensis* growth in anaerobic BC vials, no growth was evident even when we grew the high concentrations of *F. tularensis* for 15 days.

#### 3.3.3. RAST Directly from Positive Blood Culture Vials

Finally, we applied the EUCAST RAST directly from *F. tularensis*-positive BCs using the condition we found before to be optimal for *F. tularensis* RASTs, conducting both disc-diffusion- and Etest-based RASTs toward the four therapeutic antibiotics. We again determined the MIC values at the three EUCAST-recommended time points: when the BC bottles were flagged as positive, an additional 18 h incubation in the device and an additional 3 h incubation at room temperature after being positively flagged. Under these conditions, for each time point, the disc-diffusion inhibition zones were similar to those obtained with the standard *F. tularensis* inoculum (Table 15 for ciprofloxacin and Supplementary Tables S24–S26 for tetracycline, chloramphenicol and gentamicin).

Again, distinct inhibition zones could be seen following 24 h of incubation, but at least a 30-hour incubation period was required to obtain results that were similar to those of the standard reading time (48 h). Using Etest strips, RAST MIC values similar to the standard MIC values could be obtained for all antibiotics as early as 24 h of incubation (Table 16 for ciprofloxacin and Supplementary Tables S27–S30 for doxycycline, chloramphenicol, gentamycin and streptomycin), emphasizing the advantage of using Etest strips to shorten the AST duration.

## 4. Discussion

The initiation of effective antibiotic treatment is critical for treating the systemic spread of infectious bacterial pathogens. There is great importance for rapid and proper antibiotic treatment, which can increase survival rates and reduce hospitalization length and costs. Until the last few years, the standard method to perform AST assays from positive BCs required a quantified suspension of bacteria originating from agar-grown colonies. This method is time consuming and usually takes up to 2 days from the time that the BC vials were flagged positive by the automatic systems. Over the years, attempts to develop rapid AST assays for various human pathogens (for review, see [40]), including Tier 1 select agents [41,42,43], have been made. These methods can provide efficient, tailored and rapid treatment of bacterial infections. However, most of these assays are not standardized, restricting their worldwide acceptance. One of those approaches, which performs AST on a sample taken directly from a positive BC, was recently adopted by the EUCAST, the European committee responsible for promoting the standardization of AST methods used in Europe, encouraging its wide acceptance. This approach was validated for a limited number of common life-threatening bacteria that can easily acquire antibiotic resistance (*Escherichia coli*, *Klebsiella pneumoniae*, *Pseudomonas aeruginosa*, *Acinetobacter baumannii*, *Staphylococcus aureus*, *Enterococcus faecalis*, *Enterococcus faecium* and *Streptococcus pneumoniae*). However, there is a need to further extend the applicability of this method to additional pathogens.

In the current study, we examined the applicability of this direct approach to three Tier 1 select agents: *B. anthracis*, *Y. pestis* and *F. tularensis*. Due to the rapid and life-threatening clinical implications of exposure to these bacteria, together with the potential of either natural emergence or malicious generation of resistant strains in bioterror scenarios, rapid AST assays for these bacteria are highly desirable. The proficiency of the EUCAST RAST was evaluated by determining the shortest times for which proper inhibition zone diameters were derived by disc-diffusion-based AST. Using disc-diffusion assays, the assay suggested by the EUCAST guidelines, we obtained reliable inhibition zone diameters as early as 6, 18 and 24 h for *B. anthracis*, *Y. pestis* and *F. tularensis*, respectively, for most of the tested therapeutic antibiotics. However, as the inhibition diameters varied slightly from those obtained at standard reading times (18 to 20, 24 and 48 h for *B. anthracis*, *Y. pestis* and *F. tularensis*, respectively), new breakpoint interpretation tables should be established for each bacterial–antibiotic combination, as was done by EUCAST for the validated bacteria. As the establishment of new breakpoint interpretation tables is a tedious task that requires validation with multiple strains, it is rarely performed even by standard committees such as the EUCAST. We, therefore, evaluated Etest-based AST as an alternative method to determine bacterial susceptibility. We found that Etest MIC values obtained at early time points were identical to those obtained at the standard reading times; thus, category breakpoint values could be based on standard interpretation tables, such as those already published by the CLSI [38], emphasizing the advantage of using the Etest assay over disc-diffusion AST. Another advantage of Etest-based RAST is the ability to determine MIC values in addition to the susceptibility category, which can be beneficial in some clinical cases, such as in pregnant women and children and in infections of intermediate strains or when the antibiotic susceptibility breakpoint concentration is not determined. On the downside, Etest strips are relatively expensive compared to discs and, therefore, are less suitable for usage in routine clinical laboratories that perform many AST assays each day. Nevertheless, since infection with these Tier 1 select agents is not common, the financial toll for using the Etest method for these bacteria is acceptable.

One of the factors that can affect AST results is the concentration of the bacterial inoculum used in the assay. For this reason, we characterized the growth of *B. anthracis*, *Y. pestis* and *F. tularensis* in a BACTEC™ FX40 blood culture instrument, an automatic blood culturing system commonly used in clinical laboratories. This device monitors the accumulation of CO_2_ released by the growing bacteria using a fluorescent indicator present at the bottom of the BC vial and by using sophisticated algorithms to determine whether microbial growth is evident. The incubation time to positive BC alert and the bacterial concentrations at the device alert have not been previously reported for these bacteria. These data may be beneficial for the development of additional AST assays originating from grown BCs. We revealed that despite the marked difference in the bacterial concentrations that were initially spiked into the blood, when the vials were marked positive, the final concentrations were in a relatively limited range, 6.0 × 10^6^ to 8.7 × 10^7^ CFU/mL, 1.0 × 10^7^ to 5.6 × 10^7^ CFU/mL and 1.4 × 10^7^ to 3.6 × 10^8^ CFU/mL for *B. anthracis*, *Y. pestis* and *F. tularensis*, respectively. The consistency in the final bacterial concentrations was accompanied by extension of the incubation duration for blood containing low levels of bacteria. This consistency may explain the omission of the bacterial quantification step of the positive BCs before plating for RAST, according to the EUCAST guidelines.

Importantly, we found that for low levels of *F. tularensis*-spiked blood, concentrations that are still clinically relevant, the standard 5-day incubation protocol, commonly used in clinical laboratories, is not sufficiently long and may result in false-negative BCs (Table 14). Notably, when we tried to grow *F. tularensis* in anaerobic BC vials, no growth was evident, even at the highest bacterial inoculum levels, indicating that these vials do not support *F. tularensis* growth. For comparison, *B. anthracis* growth in anaerobic vials was similar to its growth in aerobic vials, and *Y. pestis* grew faster (up to 6 h) in the anaerobic vials; therefore, we used anaerobic vials to determine the RAST performance for *Y. pestis*.

Likewise, we found that the standard MH-F agar plates, which are recommended by the EUCAST guidelines for fastidious bacteria, did not support the growth of *F. tularensis* and, therefore, were not suitable for RAST or standard AST assays of *F. tularensis*. However, using CHA plates, a common medium used for growing and for MIC determination of *F. tularensis* [39], enabled the use of the RAST method to accurately determine the MIC values.

For all three tested bacteria, we could postpone the initiation of RAST, even after an additional 18 h of incubation in the device, without affecting the RAST results, suggesting that the assay can be conducted at convenient working hours in a clinical laboratory. Proper RAST results were also obtained after a delay of 3 h at room temperature of positive-flagged vials, allowing convenient transfer of the vials from one site to another.

Our results highlight the importance of validating the RAST assays with the relevant bacteria-antibiotic combinations before applying them to new bacteria. However, due to the lack of relevant strains (sensitive and resistant), we could not extensively validate the assay using more strains. This should be done by any laboratories wishing to implement this assay or by a central organization such as the EUCAST or the CLSI.

## 5. Conclusions

We have shown here that the EUCAST RAST method can be applied, with some modification, to the three Tier 1 select agents—*B. anthracis*, *Y. pestis* and *F. tularensis*—toward a panel of therapeutic antibiotics, when conducting the AST assays directly from positive BCs. The elimination of the preliminary enrichment step and shortening of the AST incubation time in the RAST assay compared to the standard AST assay can reduce labor, enable shorter time to result and most importantly lead to prompt adequate antibiotic treatment for these dangerous pathogens.

## Figures and Tables

**Table 1:** Antibiotics used in this study.

Antibiotics	Etest Concentration Range (µg/mL)	Cat #	Disc Concentration (µg)	Cat #
Ciprofloxacin (CIP)	0.002–32	412311	5	231657 ^a^
Doxycycline (DOX)	0.016–256	412328	-	-
Tetracycline (TET)	-	-	30	230998 ^a^
Chloramphenicol (CHL)	0.016–256	412309	30	CT0013B ^b^
Gentamicin (GEN)	0.016–256	412368	10	231227 ^a^
Streptomycin (STR)	0.064–1024	526800	-	-
Trimethoprim–Sulfamethoxazole (TRS)	0.002–32	412481	1.25/23.75	CT0052B ^b^
Amoxicillin–Clavulanic acid (AMC)	-	-	20/10	231628 ^a^
Ampicillin (AMP)	0.016–256	412253	-	-
Clarithromycin (CLA)	0.016–256	412313	-	-
Clindamycin (CLI)	0.016–256	412315	-	-
Imipenem (IMI)	0.002–32	412374	10	231644 ^a^
Levofloxacin (LEV)	-	-	5	231705 ^a^
Linezolid (LIN)	-	-	30	231761 ^a^
Moxifloxacin (MOX)	-	-	5	231757 ^a^
Penicillin G (BEN)	-	-	1 Unit	CT0152B ^b^
Rifampicin (RIF)	0.002–32	412450	-	-
Vancomycin (VAN)	0.016–256	412488	-	-

^a^ BD; ^b^ Oxoid.

**Table 2:** Disc-diffusion inhibition zones of germinated *B. anthracis* spores at various time points.

Incubation Duration (h)	Inhibition Zone Diameter (mm)							
	CIP	TET	BEN	AMC	LIN	MOX	LEV	IMI
6	21	25–26	19–20	28	21	17	17	31
8	22–25	27–28	20	30	30	17	23	34
16	26	30	21	31	28	23	25	38
18	26	30	21	32	28	23	26	39
20	26	30	21	33	28	23	26	39
23	24–26	29–30	20–21	30–33	28–30	23	26	39–40

CIP—ciprofloxacin; TET—tetracycline; BEN—penicillin G; AMC—amoxicillin–clavulanate; LIN—linezolid; MOX—moxifloxacin; LEV—levofloxacin; IMI—imipenem; the inhibition zone diameters were determined by at least three independent assays.

**Table 3:** Etest MIC values of germinated *B. anthracis* spores at various time points.

Incubation Duration (h)	MIC (µg/mL)							
	CIP	DOX	AMP	CHL	CLI	CLA	VAN	RIF
6	0.06	<0.016	0.03-0.06	4–8	0.125–0.5	0.25–0.5	0.125	0.03–0.06
8	0.06	≤0.016	0.03–0.06	4–8	0.125–0.5	0.25–0.5	0.25	0.03–0.125
10	0.06	≤0.016	0.06	4–8	0.25	0.25	0.25	0.06
16	0.06	0.016–0.06	0.06–0.125	4	0.25–0.5	0.25	1	0.25–0.5
18	0.06	0.016–0.125	0.06–0.125	4–8	0.25–0.5	0.25	1–2	0.25–0.5
20	0.06	0.06–0.125	0.06–0.125	4–8	0.25–0.5	0.25	1–2	0.5
23	0.06	0.06–0.125	0.06–0.125	8	0.25–0.5	0.25–0.5	1–2	0.25–0.5

CIP—ciprofloxacin; DOX—doxycycline; AMP—ampicillin; CHL—chloramphenicol; CLI—clindamycin; CLA—clarithromycin; VAN—vancomycin; RIF—rifampicin; the MIC values were determined by at least three independent assays.

**Table 4:** Characterization of *B. anthracis* growth in blood culture vials.

Initial Concentration in Blood (CFU/mL)	Incubation Duration until the Alert (hh: mm)	Concentration at the Alert (CFU/mL)	Concentration, 18 h after the Alert, Incubation in the Device (CFU/mL)	Concentration, 3 h after the Alert, at Room Temperature (CFU/mL)
1.0 × 10^5^	4:30 to 6:30	8.7 × 10^6^ to 2.9 × 10^7^	2.0 × 10^7^ to 7.0 × 10^7^	2.3 × 10^7^ to 5.0 × 10^7^
1.0 × 10^4^	6:30 to 7:50	8.0 × 10^6^ to 1.5 × 10^7^		
1.0 × 10^3^	7:50 to 9:40	6.0 × 10^6^ to 2.4 × 10^7^		
1.0 × 10^2^	9:40 to 10:30			
1.0 × 10^1^	10:50 to 11:50	3.8 × 10^6^ to 1.8 × 10^7^		

The values were determined by at least three independent assays.

**Table 5:** Ciprofloxacin inhibition zone diameters of *B. anthracis*-positive blood cultures.

	Inhibition Zone Diameter (mm)			
Incubation Duration (h)	Standard Culture ^a^	At the Alert	18 h after the Alert, Incubation in the Device	3 h after the Alert, at Room Temperature
6	21	20–21	20–21	18–21
8	22–25	23–25	22–24	20–22
16	26	23–26	ND	ND
18	26	23–26	ND	ND
20	26	23–26	ND	ND
23	24–26	23–26	22–25	21–24

^a^ Ciprofloxacin inhibition-zone diameter for the standard culture were taken from Table 2; ND—not determined; the diameters were determined by at least three independent assays.

**Table 6:** Etest ciprofloxacin MIC values for *B. anthracis*-positive blood cultures.

	MIC (µg/mL)			
Incubation Duration (h)	Standard Culture ^a^	At the Alert	18 h after the Alert, Incubation in the Device	3 h after the Alert, at Room Temperature
6	0.06	0.06	0.03–0.06	0.06
8	0.06	0.06	0.03–0.06	0.06
16	0.06	0.03–0.06	ND	ND
18	0.06	0.03–0.06	ND	ND
20	0.06	0.03–0.125	ND	ND
23	0.06	0.06–0.125	0.06	0.06

^a^ Ciprofloxacin MIC values for the standard culture were taken from Table 3; ND—not determined; the MIC values were determined by at least three independent assays.

**Table 7:** Disc-diffusion inhibition zones of *Y. pestis* at various time points.

Inhibition Zone	Inhibition Zone Diameter (mm)				
Incubation Duration (h)	CIP	TET	GEN	CHL	TRS
18	35–40	28	27–28	31–36	40–42
20	36–40	29–30	28–30	30–35	40–42
22	39–41	30	28–29	31–36	40–42
24	39–42	30–32	28–30	30–35	39–42

CIP—ciprofloxacin; TET—tetracycline; GEN—gentamicin; CHL—chloramphenicol; TRS—trimethoprim–sulfamethoxazole; the diameters were determined by at least three independent assays.

**Table 8:** Etest MIC values of *Y. pestis* at various time points.

	MIC (µg/mL)					
Incubation Duration (h)	CIP	DOX	GEN	CHL	TRS	STR
18	0.016	1–2	0.25–1	2–4	0.016–0.03	1–2
20	0.016	1–2	0.25–1	2–4	0.016–0.03	1–2
22	0.016–0.03	1–2	0.25–1	2–4	0.016–0.03	1–2
24	0.016–0.03	1–2	0.25–1	2–4	0.016–0.03	1–2

CIP—ciprofloxacin; DOX—doxycycline; GEN—gentamicin; CHL—chloramphenicol; TRS—trimethoprim–sulfamethoxazole; STR—streptomycin; the MIC values were determined by at least three independent assays.

**Table 9:** Characterization of *Y. pestis* growth in blood culture vials.

Initial Concentration in Blood (CFU/mL)	Incubation Duration until Alert (hh: mm)	Concentration at the Alert (CFU/mL)	Concentration, 18 h after the Alert, Incubation in the Device (CFU/mL)	Concentration, 3 h after the Alert, at Room Temperature (CFU/mL)
1.0 × 10^3^ to 2.7 × 10^3^	20:00 to 22:40	2.4 × 10^7^ to 3.0 × 10^7^	2.5 × 10^8^ to 3.2 × 10^8^	6.0 × 10^7^ to 8.0 × 10^7^
1.0 × 10^2^ to 2.3 × 10^2^	23:10 to 30:50	1.6 × 10^7^ to 5.6 × 10^7^		
1.4 × 10^1^ to 5.9 × 10^1^	24:40 to 29:10	1.0 × 10^7^ to 1.3 × 10^7^		

The values were determined by at least three independent assays.

**Table 10:** Ciprofloxacin inhibition zone diameters of *Y. pestis*-positive blood cultures.

	Inhibition Zone Diameter (mm)			
Incubation Duration (h)	Standard Conditions ^a^	At the Alert	18 h after the Alert, Incubation in the Device	3 h after the Alert, at Room Temperature
18	35–40	35–40	37–39	40–42
20	36–40	36–40	37–42	38–42
22	39–41	36–42	37–42	42
24	39–42	42	37–43	43–45

^a^ Ciprofloxacin inhibition zone diameter for the standard culture were taken from Table 7; the diameters were determined by at least three independent assays.

**Table 11:** Ciprofloxacin Etest MIC values of *Y. pestis*-positive blood cultures.

	MIC (µg/mL)			
Incubation Duration (h)	Standard Culture ^a^	At the Alert	18 h after the Alert, Incubation in the Device	3 h after the Alert, at Room Temperature
18	0.016	0.016–0.03	0.016	0.016
20	0.016	0.016–0.03	0.016–0.03	0.016–0.03
22	0.016–0.03	0.016–0.03	0.016–0.03	0.016
24	0.016–0.03	0.016	0.016–0.03	0.016

^a^ Ciprofloxacin MIC values for the standard culture were taken from Table 8; the MIC values were determined by at least three independent assays.

**Table 12:** Disc-diffusion inhibition zones of *F. tularensis* at various time points.

	Inhibition Zone Diameter (mm)			
Incubation Duration (h)	CIP	TET	CHL	GEN
24	40–42	39–40	40–41	23–25
30–40	42–44	40–41	41–43	26–27
48	43–44	40–42	40–43	27–28

CIP—ciprofloxacin; TET—tetracycline; CHL—chloramphenicol; GEN—gentamicin; the diameters were determined by at least three independent assays.

**Table 13:** Etest MIC values of *F. tularensis* at various time points.

	MIC (µg/mL)				
Incubation Duration (h)	CIP	DOX	CHL	GEN	STR
24	0.016	0.25	0.5–1	0.5–1	2–4
30–40	0.008–0.016	0.25–0.5	0.5–1	0.5–1	2–4
48	0.008–0.016	0.25–0.5	0.5	0.5–1	2–4

DOX—doxycycline; CIP—ciprofloxacin; CHL—chloramphenicol; GEN—gentamicin; STR—streptomycin; the MIC values were determined by at least three independent assays.

**Table 14:** Characterization of *F. tularensis* growth in blood cultures.

Initial Concentration in Blood (CFU/mL)	Incubation Duration until the Alert (dd: hh: mm)	Concentration at the Alert (CFU/mL)	Concentration, 18 h after the Alert, Incubation in the Device (CFU/mL)	Concentration, 3 h after the Alert, at Room Temperature (CFU/mL)
2.6 × 10^6^ to 2.9 × 10^6^	0:19:00 to 0:23:30	1.5 × 10^7^ to 3.0 × 10^8^	3.6 × 10^8^ to 2.3 × 10^9^	ND
1.0 × 10^5^ to 3.3 × 10^5^	1:09:00 to 2:06:30			
2.6 × 10^4^ to 2.8 × 10^4^	2:07:30 to 2:14:30			
2.6 × 10^3^ to 4.9 × 10^3^	3:23:00 to 4:17:00	1.4 × 10^7^ to 9.0 × 10^7^	1.4 × 10^8^ to 8.0 × 10^8^	ND
2.0 × 102 to 4.8 × 102	4:22:00 to 8:01:30	1.6 × 10^7^ to 3.6 × 10^8^	1.8 × 10^8^ to 1.6 × 10^9^	5.0 × 10^7^ to 5.6 × 10^8^
2.0 × 101 to 4.3 × 101	6:13:00 to 12:04:00			

ND—not determined; the values were determined by at least three independent assays.

**Table 15:** Ciprofloxacin inhibition zone diameters of *F. tularensis*-positive blood cultures.

	Inhibition Zone Diameter (mm)			
Incubation Duration (h)	Standard Culture ^a^	At the Alert	18 h after the Alert, Incubation in the Device	3 h after the Alert, at Room Temperature
24	40–42	40	40	40
30–40	42–44	43–44	42–43	43
48	43–44	44	43–44	44

^a^ Ciprofloxacin inhibition zone diameter results for the standard culture were taken from Table 12; the diameters were determined by at least three independent assays.

**Table 16:** Ciprofloxacin Etest MIC values of *F. tularensis*-positive blood cultures.

	MIC (µg/mL)			
Incubation Duration (h)	Standard Culture ^a^	At the Alert	18 h after the alert, Incubation in the Device	3 h after the Alert, at Room Temperature
24	0.016	0.008–0.016	0.016	0.016
30–40	0.008–0.016	0.008–0.016	0.008–0.016	0.008–0.016
48	0.008–0.016	0.008–0.016	0.008–0.016	0.008–0.016

^a^ Ciprofloxacin MIC values for the standard culture were taken from Table 13; the MIC values were determined by at least three independent assays.

## Data Availability

Not applicable.

## Supplementary Materials

The following are available online at https://www.mdpi.com/article/10.3390/microorganisms9051055/s1, Table S1: Tetracycline inhibition zone diameters of *B. anthracis*-positive blood cultures. Table S2: Penicillin G inhibition zone diameters of *B. anthracis*-positive blood cultures. Table S3: Amoxicillin–clavulanic acid inhibition zone diameters of *B. anthracis*-positive blood cultures. Table S4: Linezolid inhibition zone diameters of *B. anthracis*-positive blood cultures. Table S5: Moxifloxacin inhibition zone diameters of *B. anthracis*-positive blood cultures. Table S6: Levofloxacin inhibition zone diameters of *B. anthracis*-positive blood cultures. Table S7: Imipenem inhibition zone diameters of *B. anthracis*-positive blood cultures. Table S8: Etest doxycycline MIC values for *B. anthracis*-positive blood cultures. Table S9: Etest ampicillin MIC values for *B. anthracis*-positive blood cultures. Table S10: Etest clarithromycin MIC values for *B. anthracis*-positive blood cultures. Table S11: Etest chloramphenicol MIC values for *B. anthracis*-positive blood cultures. Table S12: Etest clindamycin MIC values for *B. anthracis*-positive blood cultures. Table S13. Etest vancomycin MIC values for *B. anthracis*-positive blood cultures. Table S14: Etest rifampicin MIC values for *B. anthracis*-positive blood cultures. Table S15: Tetracycline inhibition zone diameters of *Y. pestis*-positive blood cultures. Table S16: Gentamicin inhibition zone diameters of *Y. pestis*-positive blood cultures. Table S17: Chloramphenicol inhibition zone diameters of *Y. pestis*-positive blood cultures. Table S18: Trimethoprim–sulfamethoxazole inhibition zone diameters of *Y. pestis*-positive blood cultures. Table S19: Doxycycline Etest MIC values of *Y. pestis*-positive blood cultures. Table S20: Chloramphenicol Etest MIC values of *Y. pestis*-positive blood cultures. Table S21: Trimethoprim–sulfamethoxazole Etest MIC values of *Y. pestis*-positive blood cultures. Table S22: Gentamicin Etest MIC values of *Y. pestis*-positive blood cultures. Table S23: Streptomycin Etest MIC values of *Y. pestis*-positive blood cultures. Table S24: Tetracycline inhibition zone diameters of *F. tularensis*-positive blood cultures. Table S25: Chloramphenicol inhibition zone diameters of *F. tularensis*-positive blood cultures. Table S26: Gentamicin inhibition zone diameters of *F. tularensis*-positive blood cultures. Table S27: Doxycycline Etest MIC values of *F. tularensis*-positive blood cultures. Table S28: Chloramphenicol Etest MIC values of *F. tularensis*-positive blood cultures. Table S29: Gentamicin Etest MIC values of *F. tularensis*-positive blood cultures. Table S30: Streptomycin Etest MIC values of **F. tularensis**-positive blood cultures.
