# Supplementary material for: Evaluation of the European Committee on Antimicrobial Susceptibility Testing Guidelines for Rapid Antimicrobial Susceptibility Testing of Bacillus anthracis-, Yersinia pestis- and Francisella tularensis-Positive Blood Cultures
Source: Microorganisms. 2021 May 13;9(5):1055. doi: 10.3390/microorganisms9051055 (PMC8153291; doi:10.3390/microorganisms9051055)
Supplement: Supplementary file 1 [file microorganisms-09-01055-s001.zip › Blood Cultures_-supplementary tables_220321.pdf]

Table S1. Tetracycline inhibition zone diameters of *B. anthracis*-positive blood cultures

	Inhibition zone diameter (mm)			
Incubation duration (h)	Standard culture [a]	at the alert	18 h after the alert, incubation in the device	3 h after the alert, at room temperature
6	25-26	26-27	28	26
8	27-28	28-30	29-30	27
16	30	29-30	ND	ND
18	30	27-30	ND	ND
20	30	27-30	ND	ND
23	29-30	27-30	28-30	27-28

[a] Tetracycline inhibition zone diameters for the standard culture were taken from Table 2
ND - Not Determined
The inhibition-zone diameters were determined by at least three independent assays

Table S2. Penicillin G inhibition zone diameters of *B. anthracis*-positive blood cultures

	Inhibition zone diameter (mm)			
Incubation duration (h)	Standard culture [a]	at the alert	18 h after the alert, incubation in the device	3 h after the alert, at room temperature
6	19-20	20-22	19-21	18-19
8	20	20-22	19-21	19
16	21	20-21	ND	ND
18	21	19-21	ND	ND
20	21	19-21	ND	ND
23	20-21	19-22	19-21	18-20

[a] Penicillin G inhibition zone diameter for the standard culture were taken from Table 2
ND - Not Determined
The inhibition-zone diameters were determined by at least three independent assays

Table S3. Amoxicillin-Clavulanic acid inhibition zone diameters of *B. anthracis*-positive blood cultures

	Inhibition zone diameter (mm)			
Incubation duration (h)	Standard culture [a]	at the alert	18 h after the alert, incubation in the device	3 h after the alert, at room temperature
6	28	27-30	28-29	25-26
8	30	29-30	29-30	27-28
16	31	30-32	ND	ND
18	32	30-32	ND	ND
20	33	30-32	ND	ND
23	30-33	30-32	29-32	27-30

[a] Amoxicillin-Clavulanic acid inhibition zone diameters for the standard culture were taken from Table 2
ND - Not Determined
The inhibition-zone diameters were determined by at least three independent assays

Table S4. Linezolid inhibition zone diameters of *B. anthracis*-positive blood cultures

	Inhibition zone diameter (mm)			
Incubation duration (h)	Standard culture [a]	at the alert	18 h after the alert, incubation in the device	3 h after the alert, at room temperature
6	21	22-25	26-28	26-27
8	30	28-30	26-28	26-27
16	28	29-30	ND	ND
18	28	28-30	ND	ND
20	28	28-30	ND	ND
23	28-30	28-32	27-30	26-28

[a] Linezolid inhibition zone diameter for the standard culture were taken from Table 2
ND - Not Determined
The inhibition-zone diameters were determined by at least three independent assays

Table S5. Moxifloxacin inhibition zone diameters of *B. anthracis*-positive blood cultures

	Inhibition zone diameter (mm)			
**Incubation duration (h)**	**Standard culture [a]**	**at the alert**	**18 h after the alert, incubation in the device**	**3 h after the alert, at room temperature**
6	17	17-19	18-19	13-17
8	17	20-21	19-20	17-18
16	23	21-23	ND	ND
18	23	21-23	ND	ND
20	23	21-23	ND	ND
23	23	21-23	19-22	19

[a] Moxifloxacin inhibition zone diameter for the standard culture were taken from Table 2
ND - Not Determined
The inhibition-zone diameters were determined by at least three independent assays

Table S6. Levofloxacin inhibition zone diameters of *B. anthracis*-positive blood cultures

	Inhibition zone diameter (mm)			
**Incubation duration (h)**	**Standard culture [a]**	**at the alert**	**18 h after the alert, incubation in the device**	**3 h after the alert, at room temperature**
6	17	19-20	20	18-20
8	23	23-24	20-25	18-20
16	25	24-25	ND	ND
18	26	24-26	ND	ND
20	26	24-26	ND	ND
23	26	25-26	22-25	19-22

[a] Levofloxacin inhibition zone diameter results for the standard culture were taken from Table 2
ND - Not Determined
The inhibition-zone diameters were determined by at least three independent assays

Table S7. Imipenem inhibition zone diameters of *B. anthracis*-positive blood cultures

	Inhibition zone diameter (mm)			
**Incubation duration (h)**	**Standard culture [a]**	**at the alert**	**18 h after the alert, incubation in the device**	**3 h after the alert, at room temperature**
6	31	30	31-32	30-31
8	34	33-36	32-35	33
16	38	37-39	ND	ND
18	39	37-39	ND	ND
20	39	37-39	ND	ND
23	39-40	38-42	37-40	35

[a] Imipenem inhibition zone diameters results for the standard culture were taken from Table 2
ND - Not Determined
The inhibition-zone diameters were determined by at least three independent assays

Table S8. Etest Doxycycline MIC values for *B. anthracis* positive blood cultures

	MIC (μg/ml)			
**Incubation duration (h)**	**Standard culture [a]**	**at the alert**	**18 h after the alert, incubation in the device**	**3 h after the alert, at room temperature**
6	<0.016	≤0.016	0.016	0.016
8	≤0.016	≤0.016	0.016	0.016
16	0.016-0.06	0.03-0.06	ND	ND
18	0.016-0.125	0.03-0.06	ND	ND
20	0.06-0.125	0.03-0.06	ND	ND
23	0.06-0.125	0.06-0.125	0.06-0.125	0.06

[a] Doxycycline MIC values for the standard culture were taken from Table 3
ND - Not Determined
The MIC values were determined by at least three independent assays

Table S9. Etest Ampicillin MIC values for *B. anthracis* positive blood cultures

	MIC (μg/ml)			
**Incubation duration (h)**	**Standard culture [a]**	**at the alert**	**18 h after the alert, incubation in the device**	**3 h after the alert, at room temperature**
6	0.03-0.06	0.03-0.125	0.06	0.06
8	0.03-0.06	0.03-0.06	0.06	0.06
16	0.06-0.125	0.06	ND	ND
18	0.06-0.125	0.06	ND	ND
20	0.06-0.125	0.06	ND	ND
23	0.06-0.125	0.06	0.125	0.125

[a] Ampicillin MIC values for the standard culture were taken from Table 3
ND - Not Determined
The MIC values were determined by at least three independent assays

Table S10. Etest Clarithromycin MIC values for *B. anthracis* positive blood cultures

	MIC (μg/ml)			
**Incubation duration (h)**	**Standard culture [a]**	**at the alert**	**18 h after the alert, incubation in the device**	**3 h after the alert, at room temperature**
6	0.25-0.5	0.125-0.25	0.25	0.25
8	0.25-0.5	0.125-0.25	0.125-0.25	0.25
16	0.25	0.25	ND	ND
18	0.25	0.25	ND	ND
20	0.25	0.25	ND	ND
23	0.25-0.5	0.125-0.25	0.25-0.5	0.5

[a] Clarithromycin MIC values for the standard culture were taken from Table 3
ND - Not Determined
The MIC values were determined by at least three independent assays

Table S11. Etest Chloramphenicol MIC values for *B. anthracis* positive blood cultures

	MIC (μg/ml)			
**Incubation duration (h)**	**Standard culture [a]**	**at the alert**	**18 h after the alert, incubation in the device**	**3 h after the alert, at room temperature**
6	4-8	2-4	2-4	4
8	4-8	2-4	2-4	4-8
16	4	4-8	ND	ND
18	4-8	4-8	ND	ND
20	4-8	8	ND	ND
23	8	4-8	4-8	4-8

[a] Chloramphenicol MIC values for the standard culture were taken from Table 3
ND - Not Determined
The MIC values were determined by at least three independent assays

Table S12. Etest Clindamycin MIC values for *B. anthracis* positive blood cultures

	MIC (μg/ml)			
**Incubation duration (h)**	**Standard culture [a]**	**at the alert**	**18 h after the alert, incubation in the device**	**3 h after the alert, at room temperature**
6	0.125-0.5	0.06-0.125	0.25	0.125
8	0.125-0.5	0.125	0.125-0.25	0.125
16	0.25-0.5	0.25	ND	ND
18	0.25-0.5	0.125	ND	ND
20	0.25-0.5	0.125	ND	ND
23	0.25-0.5	0.125-0.25	0.125-0.25	0.125

[a] Clindamycin MIC values for the standard culture were taken from Table 3
ND - Not Determined
The MIC values were determined by at least three independent assays

Table S13. Etest Vancomycin MIC values for *B. anthracis* positive blood cultures

Incubation duration (h)	MIC (µg/ml)	
	**Standard culture [a]**	**at the alert**
6	0.125	ND
8	0.25	ND
16	1	1-2
18	1-2	1-2
20	1-2	1-2
23	1-2	1-2

[a] Vancomycin MIC values for the standard culture were taken from Table 3
ND - Not Determined
The MIC values were determined by at least three independent assays

Table S14. Etest Rifampicin MIC values for *B. anthracis* positive blood cultures

Incubation duration (h)	MIC (µg/ml)	
	**Standard culture [a]**	**at the alert**
6	0.03-0.06	ND
8	0.03-0.125	ND
16	0.25-0.5	0.25
18	0.25-0.5	0.25
20	0.5	0.25
23	0.25-0.5	0.25

[a] Rifampicin MIC values for the standard culture were taken from Table 3
ND-Not Determined
The MIC values were determined by at least three independent assays

Table S15. Tetracycline inhibition zone diameters of *Y. pestis*-positive blood cultures

Incubation duration (h)	Inhibition zone diameter (mm)			
	**Standard culture [a]**	**at the alert**	**18 h after the alert, incubation in the device**	**3 h after the alert, at room temperature**
18	28	27-29	29-30	27-29
20	29-30	30	30-32	28-29
22	30	29-30	30	29
24	30-32	31	30-31	29-30

[a] Tetracycline inhibition zone diameters for the standard culture were taken from Table 7
The inhibition zone diameters were determined by at least three independent assays

Table S16. Gentamicin inhibition zone diameters of *Y. pestis*-positive blood cultures

Incubation duration (h)	Inhibition zone diameter (mm)			
	**Standard culture [a]**	**at the alert**	**18 h after the alert, incubation in the device**	**3 h after the alert, at room temperature**
18	27-28	26-29	27-30	27-29
20	28-30	26-30	27-30	27-29
22	28-29	27-30	28-30	27-29
24	28-30	30	28-30	28-29

[a] Gentamicin inhibition zone diameters for the standard culture were taken from Table 7
The inhibition-zone diameters were determined by at least three independent assays

Table S17. Chloramphenicol inhibition zone diameters of *Y. pestis*-positive blood cultures

Incubation duration (h)	Inhibition zone diameter (mm)			
	**Standard culture [a]**	**at the alert**	**18 h after the alert, incubation in the device**	**3 h after the alert, at room temperature**
18	31-36	34-35	32-35	33-34
20	30-35	35	35	34
22	31-36	32-35	35	31
24	30-35	35	35	31-35

[a] Chloramphenicol inhibition zone diameters for the standard culture were taken from Table 7
The inhibition-zone diameters were determined by at least three independent assays

Table S18. Trimethoprim-Sulfamethoxazole inhibition zone diameters of *Y. pestis*-positive blood cultures

	Inhibition zone diameter (mm)			
**Incubation duration (h)**	**Standard culture [a]**	**at the alert**	**18 h after the alert, incubation in the device**	**3 h after the alert, at room temperature**
18	40-42	41-42	39-43	40-43
20	40-42	42-43	39-43	40-43
22	40-42	42-44	39-43	40-43
24	39-42	44	40-43	40-43

[a] Trimethoprim-Sulfamethoxazole inhibition zone diameters for the standard culture were taken from Table 7
The inhibition-zone diameters were determined by at least three independent assays

Table S19. Doxycycline Etest MIC values of *Y. pestis*-positive blood cultures

	MIC (µg/ml)			
**Incubation duration (h)**	**Standard culture [a]**	**at the alert**	**18 h after the alert, incubation in the device**	**3 h after the alert, at room temperature**
18	1-2	1-2	1-2	1-2
20	1-2	1-2	1-2	1-2
22	1-2	1-2	1-2	1-2
24	1-2	1	1-2	1-2

[a] Doxycycline MIC values for the standard culture were taken from Table 8
The MIC values were determined by at least three independent assays

Table S20. Chloramphenicol Etest MIC values of *Y. pestis*-positive blood cultures

	MIC (µg/ml)			
**Incubation duration (h)**	**Standard culture [a]**	**at the alert**	**18 h after the alert, incubation in the device**	**3 h after the alert, at room temperature**
18	2-4	2	2-4	2-4
20	2-4	2	2-4	2-4
22	2-4	2	2-4	2-4
24	2-4	2	2-4	4

[a] Chloramphenicol MIC values for the standard culture were taken from Table 8
The MIC values were determined by at least three independent assays

Table S21. Trimethoprim-Sulfamethoxazole Etest MIC values of *Y. pestis*-positive blood cultures

	MIC (µg/ml)			
**Incubation duration (h)**	**Standard culture [a]**	**at the alert**	**18 h after the alert, incubation in the device**	**3 h after the alert, at room temperature**
18	0.016-0.03	0.016-0.03	0.016-0.03	0.016-0.03
20	0.016-0.03	0.016-0.03	0.016-0.03	0.016-0.03
22	0.016-0.03	0.016-0.03	0.016-0.03	0.016-0.03
24	0.016-0.03	0.016	0.016-0.03	0.016-0.03

[a] Trimethoprim-Sulfamethoxazole MIC values for the standard culture were taken from Table 8
The MIC values were determined by at least three independent assays

Table S22. Gentamicin Etest MIC values of *Y. pestis*-positive blood cultures

	MIC (µg/ml)			
**Incubation duration (h)**	**Standard culture [a]**	**at the alert**	**18 h after the alert, incubation in the device**	**3 h after the alert, at room temperature**
18	0.25-1	0.5	0.25-1	0.25-0.5
20	0.25-1	0.5	0.5	0.25-0.5
22	0.25-1	0.5	0.5	0.25-0.5
24	0.25-1	0.25	0.5	0.25-0.5

[a] Gentamicin MIC values for the standard culture were taken from Table 8
The MIC values were determined by at least three independent assays

Table S23. Streptomycin Etest MIC values of *Y. pestis*-positive blood cultures cultures

Incubation duration (h)	MIC (μg/ml)			
	Standard culture [a]	at the alert	18 h after the alert, incubation in the device	3 h after the alert, at room temperature
18	1-2	2	1-2	1-2
20	1-2	2	1-2	1-2
22	1-2	2	1-2	1-2
24	1-2	1	1-2	1-2

[a] Streptomycin MIC values for the standard culture were taken from Table 8
The MIC values were determined by at least three independent assays

Table S24. Tetracycline inhibition zone diameters of *F. tularensis*-positive blood cultures

Incubation duration (h)	Inhibition zone diameter (mm)			
	Standard culture [a]	at the alert	18 h after the alert, incubation in the device	3 h after the alert, at room temperature
24	39-40	40	39-40	38-40
30-40	40-41	40	40	40
48	40-42	40-41	40-41	40-41

[a] Tetracycline inhibition zone diameters results for the standard culture were taken from Table 12
The inhibition zone diameters were determined by at least three independent assays

Table S25. Chloramphenicol inhibition zone diameters of *F. tularensis*-positive blood cultures

Incubation duration (h)	Inhibition zone diameter (mm)			
	Standard culture [a]	at the alert	18 h after the alert, incubation in the device	3 h after the alert, at room temperature
24	40-41	40-41	40	40
30-40	41-43	40-43	41-42	41-43
48	40-43	41-43	41-42	41-43

[a] Chloramphenicol inhibition zone diameters for the standard culture were taken from Table 12
The inhibition zone diameters were determined by at least three independent assays

Table S26. Gentamicin inhibition zone diameters of *F. tularensis*-positive blood cultures

Incubation duration (h)	Inhibition zone diameter (mm)			
	Standard culture [a]	at the alert	18 h after the alert, incubation in the device	3 h after the alert, at room temperature
24	23-25	23-25	24-26	23-25
30-40	26-27	26-27	26-27	26-27
48	27-28	28	27-28	28

[a] Gentamicin inhibition zone diameters for the standard culture were taken from Table 12
The inhibition zone diameters were determined by at least three independent assays

Table S27. Doxycycline Etest MIC values of *F. tularensis*-positive blood cultures

Incubation duration (h)	MIC (μg/ml)			
	Standard culture [a]	at the alert	18 h after the alert, incubation in the device	3 h after the alert, at room temperature
24	0.25	0.25-0.5	0.25	0.25-0.5
30-40	0.25-0.5	0.25-0.5	0.25-0.5	0.25-0.5
48	0.25-0.5	0.5	0.25-0.5	0.25-0.5

[a] Doxycycline MIC values for the standard culture were taken from Table 13
The MIC values were determined by at least three independent assays

Table S28. Chloramphenicol Etest MIC values of *F. tularensis*-positive blood cultures

	MIC (μg/ml)			
**Incubation duration (h)**	**Standard culture [a]**	**at the alert**	**18 h after the alert, incubation in the device**	**3 h after the alert, at room temperature**
24	0.5-1	0.5	0.5-1	0.5-1
30-40	0.5-1	0.5	0.5	0.5
48	0.5	0.5	0.5-1	0.5-1

[a] Chloramphenicol MIC values for the standard culture were taken from Table 13
The MIC values were determined by at least three independent assays

Table S29. Gentamicin Etest MIC values of *F. tularensis*-positive blood cultures

	MIC (μg/ml)			
**Incubation duration (h)**	**Standard culture [a]**	**at the alert**	**18 h after the alert, incubation in the device**	**3 h after the alert, at room temperature**
24	0.5-1	0.5-1	1	1
30-40	0.5-1	0.25-1	0.5-1	0.5-1
48	0.5-1	0.25-1	0.5-1	0.5

[a] Gentamicin MIC values for the standard culture were taken from Table 13
The MIC values were determined by at least three independent assays

Table S30. Streptomycin Etest MIC values of *F. tularensis*-positive blood cultures

	MIC (μg/ml)			
**Incubation duration (h)**	**Standard culture [a]**	**at the alert**	**18 h after the alert, incubation in the device**	**3 h after the alert, at room temperature**
24	2-4	2-4	2-4	2
30-40	2-4	1-4	2-4	1-2
48	2-4	1-4	2-4	1-2

[a] Streptomycin MIC values for the standard culture were taken from Table 13
The MIC values were determined by at least three independent assays
